# Effective strategies for scaling up evidence-based practices in primary care: a systematic review

**DOI:** 10.1186/s13012-017-0672-y

**Published:** 2017-11-22

**Authors:** Ali Ben Charif, Hervé Tchala Vignon Zomahoun, Annie LeBlanc, Léa Langlois, Luke Wolfenden, Sze Lin Yoong, Christopher M. Williams, Roxanne Lépine, France Légaré

**Affiliations:** 1 Health and Social Services Systems, Knowledge Translation and Implementation component of the Quebec SPOR-SUPPORT Unit, Université Laval, Quebec, QC Canada; 2 Tier 1 Canada Research Chair in Shared Decision Making and Knowledge Translation, Université Laval, Quebec, QC Canada; 3 Centre de recherche sur les soins et les services de première ligne (CERSSPL), Université Laval, Quebec, QC Canada; 4 Department of Family Medicine and Emergency Medicine, Université Laval, Quebec, QC Canada; 5 Population Health and Practice-Changing Research Group, CHU de Québec Research Centre, Quebec, QC Canada; 6 School of Medicine and Public Health, The University of Newcastle, Callaghan, NSW 2308 Australia; 7 Hunter Medical Research Institute, New Lambton Heights, NSW 2305 Australia; 8 Hunter New England Population Health, Wallsend, NSW 2287 Australia; 9 Centre de recherche sur les soins et les services de première ligne de l’Université Laval (CERSSPL-UL), Pavillon Landry-Poulin - 2525 Chemin de la Canardière, Quebec City, QC G1J 0A4 Canada

**Keywords:** Primary care, Knowledge translation, Scaling up, Spread, Evidence-based practices, Systematic review, Implementation

## Abstract

**Background:**

While an extensive array of existing evidence-based practices (EBPs) have the potential to improve patient outcomes, little is known about how to implement EBPs on a larger scale. Therefore, we sought to identify effective strategies for scaling up EBPs in primary care.

**Methods:**

We conducted a systematic review with the following inclusion criteria: (i) *study design*: randomized and non-randomized controlled trials, before-and-after (with/without control), and interrupted time series; (ii) *participants*: primary care-related units (e.g., clinical sites, patients); (iii) *intervention*: any strategy used to scale up an EBP; (iv) c*omparator*: no restrictions; and (v) *outcomes*: no restrictions. We searched MEDLINE, Embase, PsycINFO, Web of Science, CINAHL, and the Cochrane Library from database inception to August 2016 and consulted clinical trial registries and gray literature. Two reviewers independently selected eligible studies, then extracted and analyzed data following the Cochrane methodology. We extracted components of scaling-up strategies and classified them into five categories: infrastructure, policy/regulation, financial, human resources-related, and patient involvement. We extracted scaling-up process outcomes, such as coverage, and provider/patient outcomes. We validated data extraction with study authors.

**Results:**

We included 14 studies. They were published since 2003 and primarily conducted in low-/middle-income countries (*n* = 11). Most were funded by governmental organizations (*n* = 8). The clinical area most represented was infectious diseases (HIV, tuberculosis, and malaria, *n* = 8), followed by newborn/child care (*n* = 4), depression (*n* = 1), and preventing seniors’ falls (*n* = 1). Study designs were mostly before-and-after (without control, *n* = 8). The most frequently targeted unit of scaling up was the clinical site (*n* = 11). The component of a scaling-up strategy most frequently mentioned was human resource-related (*n* = 12). All studies reported patient/provider outcomes. Three studies reported scaling-up coverage, but no study quantitatively reported achieving a coverage of 80% in combination with a favorable impact.

**Conclusions:**

We found few studies assessing strategies for scaling up EBPs in primary care settings. It is uncertain whether any strategies were effective as most studies focused more on patient/provider outcomes and less on scaling-up process outcomes. Minimal consensus on the metrics of scaling up are needed for assessing the scaling up of EBPs in primary care.

**Trial registration:**

This review is registered as PROSPERO CRD42016041461.

**Electronic supplementary material:**

The online version of this article (10.1186/s13012-017-0672-y) contains supplementary material, which is available to authorized users.

## Background

Primary care is a critical component of the health system and is at the heart of important reforms [1–3]. Evidence shows that countries with a strong primary care component in their health system have better health outcomes and are better at keeping costs under control [4, 5]. Primary care delivery is also associated with lower mortality rates and a more equitable distribution of health in populations than is specialty care [3]. In addition, primary care reaches more people. In Canada, for example, more than three times as many people with chronic diseases contact a primary care physician than contact a specialist [6]. Unfortunately, despite its importance, the provision of primary care consistent with the exponentially increasing evidence is variable. Evidence-practice gaps are a concern and reduce the potential benefit of primary care health services to the community [7, 8].

The development of evidence-based practices (EBPs) generally follows several steps including the testing of the practice under optimal conditions (efficacy trials), followed by testing in real-world conditions (effectiveness trials) [9]. There have been many efforts to generate evidence and determine the degree of rigor required to qualify practices and programs as *evidence-based* [10]. This has resulted in an extensive array of new ideas, products, services, care, tools, programs, and policies whose evidence base has been rigorously established. However, there is a widespread failure to extend these practices to larger populations. Scaling up EBPs to primary care contexts specifically could benefit the quality of care for a larger number of individuals. In order for EBPs to realize their full benefits in primary care, there is a need to understand how they can move from single trials of local innovative projects to broad-scale use.

Although scaling up is an acknowledged concept in the field of knowledge translation and implementation science, most publications focus on methods or strategies to enhance the uptake, implementation, or sustainability of EBPs, while leaving out the step of scaling up the implementation to enable the EBPs to benefit whole populations [11–13]. A clear definition of scaling up has not emerged in the literature, nor have many theories, frameworks, or strategies been proposed to support its implementation [14]. The term “spread” is commonly used interchangeably with “scale up” [14–16]. However, while “spread” suggests the organic process of the diffusion of a local improvement within a health system [17], “scale up” implies a systematic approach often used in the context of rolling out a successful local program to regional, national, or international levels [14].

As we were not aware of any systematic reviews (completed or in progress), we sought to identify effective strategies for scaling up EBPs in primary care.

## Methods

We conducted a systematic review, following the methodology suggested by the Cochrane Effective Practice and Organisation of Care (EPOC) Review Group [18]. We reported our findings according to the Preferred Reporting Items for Systematic Reviews and Meta-Analyses (PRISMA) Statement [19, 20]. The review is registered in the International Prospective Register of Systematic Reviews (PROSPERO, CRD42016041461) [21].

### Criteria for considering studies for this review

Our specific research question was: “What are effective strategies for the scaling up of EBPs in primary care?” Following the PICO approach (participants, intervention, comparator, outcome), we used the following criteria: *participants* (*P*)—any individual, organization, or system involved in the delivery or receipt of primary health services that was the target of the scale-up of an EBP; *intervention* (*I*)—any component of a strategy used to scale up an EBP; *comparator* (*C*)—no restrictions; and *outcomes* (*O*)—any outcomes (i.e., no restriction), including measures associated with the scaling-up process (e.g., number of targeted units that benefited from the use of an EBP out of all of those targeted) and provider- or patient-reported outcomes regarding the effect of the EBP (Additional file 1).

#### Types of participants

We included any individual, organization, or system involved in the delivery or receipt of primary health services (e.g., geographical regions, clinical sites, policymakers/managers, health care providers, patients) that were the target of the scale-up of an EBP. We refer to these targeted participants as the “units” that the EBP was intended to reach (i.e., intended recipients). For example, an EBP could be implemented in several clinical sites to cover a certain number of patients; we identified both as units. Furthermore, we defined primary care, in the context of this review, as the level of the health system that provides individuals with (1) the first point of access to the system for all their health needs and problems; (2) care for all but very uncommon or unusual conditions; (3) continuity of care; and (4) the coordination or integration of the care provided by other levels of the system or by other professionals [22, 23]. Primary and secondary care each have their own cultures, policies, and traditions, and a clear difference between implementation and impact issues in these two settings has been identified [24]. As we were not aware of strategies to scale EBPs in any medical setting [25], we chose to identify scaling-up strategies potentially useful and transferable to primary care settings. Scaling-up efforts in this setting are likely to impact larger populations, are more likely to increase equity, and have more important health outcomes [3–6].

#### Types of interventions and comparators

We included any component of a strategy used to scale up an EBP. In the context of this review, a scaling-up strategy refers to any process that aims to expand the coverage of an EBP (i.e., a practice that has been reported successful by the authors) to multiple settings (or targeted primary care units). We drew a distinction between the components of strategies used to expand an EBP (i.e., scaling-up strategies, our main focus as the intervention of interest) and the components of the EBP itself [9, 26].

Combining Cochrane’s EPOC guidelines with various published scaling-up guides [16, 27–29], we identified the five following components of scaling-up strategies: (C_1_) healthcare infrastructure-related (e.g., providing medical equipment or changing linkages within a health system), (C_2_) policy and regulation-related (e.g., revising policy to allow widespread community-based case management of a disease), (C_3_) financing-related (e.g., changing payment mechanisms), (C_4_) human resource-related (e.g., training and deployment of health care providers, changing roles of administrators), and (C_5_) patient-related (e.g., involving patients/public in recruitment or promotion). We defined a vertical scaling-up strategy as the expansion of an EBP simultaneously to a whole system (as a result of a change of national policy, for example), while a horizontal scaling-up strategy referred to the expansion of an EBP across different settings in a phased manner [16, 27].

Finally, the comparator consisted of one or more alternative scaling-up strategies or usual practice (i.e., no restrictions).

#### Outcomes

We considered a wide range of outcomes (i.e., no restrictions). First, we considered measures associated with the scaling-up process, such as the coverage of the targeted units and cost of scaling up the EBP. Within the concept of coverage, some distinguish between “reach” (individuals targeted who were reached by the intervention) and “adoption” (institutions targeted that adopted the intervention) [27, 30–32]. In keeping with a recent study by Fixsen et al., we defined coverage as the number of the targeted units (individuals, organizations, or systems) that were benefiting from the use of the EBP (i.e., the numerator for the scaling up) divided by the total number of targeted units (i.e., the denominator for the scaling up) [33]. Second, we also considered any reported outcomes at the level of the health system (e.g., clinical site performance) as well as provider- and patient-reported outcomes regarding the effect of the EBPs.

#### Types of studies

We considered randomized and non-randomized controlled trials, controlled before-and-after, before-and-after (i.e., pre-post with no control group), and interrupted time series (with at least three data points before and after the scaling up). We excluded literature reviews and meta-analyses. Finally, there were no restrictions on length of study follow-up, language of publication, or country of origin.

### Literature search

Our information specialist (RL) performed the search strategy to identify published studies in the following electronic bibliographic databases (from inception to 30th August 2016): MEDLINE (Ovid and PubMed), Embase, PsycINFO, Web of Science, CINAHL, and the Cochrane Library. The search terms were developed in consultation with the other authors using a combination of keywords and Medical Subject Headings (MeSH). We also followed some recommendations of a previous review about terms to use for scaling up [15]. The search strategy was first developed in MEDLINE (Ovid) (Additional file 2) and was adapted to the other databases. We used keywords such as “scaling up,” “scalability,” or “spread.” We used an adapted filter for limiting the search to studies conducted in primary care settings [34] and a study design filter. All search results were imported into EndNote X7. Duplicate search results were identified by the software and were eliminated using a method that enables retaining unique citations without accidentally excluding false duplicates [35].

We also conducted searches in clinical trial registries and identified gray literature using the search engines and websites of relevant organizations (Additional file 3). We searched published bibliographies of related topics and citations in included articles, and contacted experts in the field and relevant organizations in Canada (e.g., Canadian Institutes of Health Research, Primary Health Care Innovation Teams [36]).

### Data collection

#### Study selection

We developed a study selection form based on our eligibility criteria. After removal of duplicates, two review authors (ABC, LL) independently piloted the study selection form with a small random sample of studies to assess understanding of eligibility criteria and ease of use of the form. Two review authors independently screened all titles/abstracts and full text to identify the relevant studies. For all ineligible studies, we documented the main reason for exclusion. Discrepancies between review authors regarding study eligibility were resolved by consensus or, when required, with a third party (FL, HTVZ).

#### Data extraction

We developed a data extraction form using a guide for scaling up [27, 37] and the Cochrane EPOC resources [18]. Two review authors (ABC, LL) independently extracted characteristics from the included studies: year of publication, the country’s economic status (low-, middle-, or high-income), funding source, clinical area, study design, setting, name of EBP, and PICO elements. We also extracted the number of components of (multifaceted) scaling-up strategies mentioned in each study, the number of units targeted, the number of units covered, the timeframe of the scaling-up process and the frameworks/theories used. We validated data extraction of eligible studies with their authors by email with a reminder through *ResearchGate* (Additional file 4). We resolved any disagreement in the data collection process through discussion and consensus between the two reviewers and, if needed, with a third party (FL, HTVZ).

#### Quality assessment

Two independent reviewers completed the quality assessment of each included study using the Quality Assessment Tool for Quantitative Studies developed by the Effective Public Health Practice Project [38]. This generic tool is used to evaluate a variety of intervention study designs such as randomized and non-randomized controlled studies, controlled before-and-after studies, and uncontrolled studies. The tool has been assessed for content and construct validity and meets accepted standards [38]. In addition, unlike the Cochrane Collaboration Risk of Bias Tool, the tool performs with high interrater reliability [39]. It assesses six domains: selection bias, study design, confounders, blinding, data collection method, and withdrawals/dropouts. Each domain was rated “strong,” “moderate,” or “weak.” Studies were assigned a quality rating of strong (if no domain was rated weak), moderate (if one domain was rated weak), or weak (if at least two domains were rated weak). All disagreements were resolved through discussion between the two reviewers and a third party.

### Data analysis

We used the PRISMA flowchart to describe the process of study selection [40]. The nature of our question, along with the lack of a standard methodological approach for evaluating scaling-up strategies, meant that it was not feasible to perform a meta-analysis [41]. Thus, data extracted from the included studies were analyzed using simple frequency counts and a narrative approach [42]. We described general characteristics of included studies, participants, components of scaling-up strategies, successful coverage of the targeted units, and impact of the strategies. We defined a successful scaling-up strategy as one which achieved 80% of the intended coverage of targeted units with a favorable impact on the main outcomes of the study. This coverage threshold is defined in several international scaling-up initiatives, including Avahan (India) [43–45], Reaching Every District (World Health Organization) [46–48], and the US President’s Emergency Plan for AIDS Relief [49]. In addition, the 80% threshold was the saturation level used in a synthesis of over 500 studies on the impact of implementation processes [50]. Finally, we also described the quality assessment of all included studies.

## Results

### Study selection

**Fig. 1:**
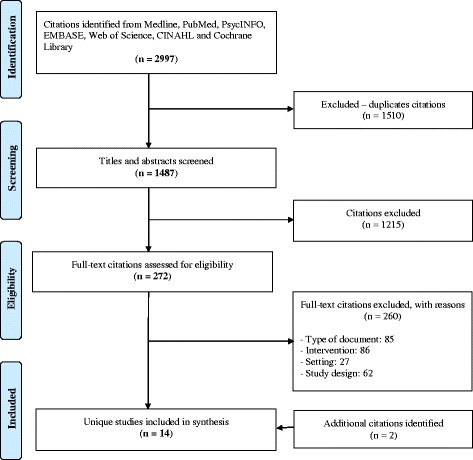
PRISMA flow diagram of the study inclusion process

Our electronic search identified 2997 potentially relevant studies. Of these, 1510 were duplicates, leaving 1487 studies. Of these, 1215 did not meet the review criteria. Thus, we reviewed a total of 272 full-text papers and retained 12. A second search (author contacts and gray literature) led to the inclusion of two additional studies. Overall, a total of 14 unique studies were included in this review [51–64] (Fig. 1).

### Characteristics of included studies

Characteristics of included studies are outlined in Table 1. All included studies were published since 2003. The majority were conducted in middle-income countries (*n* = 6) [51, 53, 58, 59, 61, 64], followed by low-income countries (*n* = 5) [52, 54, 55, 60, 63] and high-income countries (*n* = 3) [56, 57, 62]. They were mostly funded by international (e.g., United Nations) and/or national governmental organizations (*n* = 8) [51–54, 57, 59, 60, 63], followed by voluntary or charitable bodies (*n* = 3) [55, 61, 64] and research bodies (*n* = 1) [56]. For two studies [58, 62], this information was not found. Most EBPs concerned preventing or treating infectious diseases: HIV, tuberculosis, and malaria (*n* = 8) [51–54, 56, 58, 59, 61]. Others concerned newborn/child care (*n* = 4) [55, 60, 63, 64], depression (*n* = 1) [62], and preventing seniors’ falls (*n* = 1) [57].

**Table 1:** Characteristics of included studies (*n* = 14)

Reference in chronological order	General characteristics						Targeted units	Component of scaling-up strategy^a^					Outcomes	Outcomes		
	Country economic status	Funding source	Clinical area	Study design	Setting	Evidence-based practice		C_1_	C_2_	C_3_	C_4_	C_5_	Scaling-up process^b^	Health system	Provider	Patient
Frieden et al. [51]	MIC	Governmental organization	Tuberculosis	Non-RCT	State and district health tuberculosis units	Treatment, short-course (DOTS) strategy for tuberculosis control	Region, patient				⊠		Coverage			Sputum smear conversion, cure rate; treatment success
Price et al. [52]	LIC	Governmental organization	HIV	Before-after	Primary health centers	Basic HIV care	Site		⊠							Reproductive health, services for children, curative services
Mutevedzi et al. [53]	MIC	Governmental organization	HIV	Before-after	Primary health care clinics	HIV treatment and care program	Site, patient				⊠					Retention in care, mortality, loss to follow-up and virological outcomes
Renju et al. [54]	LIC	Governmental organization	Sexually transmitted infection and reproductive health	Before-after	Health units (hospitals, health centers and dispensaries)	Youth-friendly services (YES) intervention	Site, provider				⊠				Knowledge and attitudes of health workers	
Curry et al. [55]	LIC	Voluntary/charitable body	Child care	Before-after	Primary health care units	Some items of Millennium Rural Initiative (EMRI)	Site	⊠			⊠		Cost-benefit ratio of the EMRI program	Health centre infrastructure and performance		Maternal and child survival
Goetz et al. [56]	HIC	Research funding body	HIV	Non-RCT	Veterans' healthcare administration facilities	A multimodal program to promote HIV testing	Site		⊠		⊠			Semi-annual patient load per facility, number of patient visits per year, prevalence of HIV, complexity level	Number of providers seeing the patients during the 6 months of routine testing	HIV testing
Li et al. [57]	HIC	Governmental organization	Preventing seniors' falls	Before-after	Local senior and community centers	Tai Ji Quan: Moving for Better Balance	Provider				⊠		Coverage; measures of program implementation, maintenance and effectiveness			Body mass index, incidence of falls, fear of falling, health status, number of chronic medical conditions
Miyano et al. [58]	MIC	Not found	Tuberculosis	CBA	Hospitals and rural health centers	Antiretroviral therapy (ART) services program	Site	⊠			⊠					HIV testing, tuberculosis treatment outcomes (success, died/failed)
Comfort et al. [59]	MIC	Governmental organization	Malaria	Before-after	Hospitals: rural health centers and primary health centers	Malaria control interventions	Site, patient		⊠		⊠			Costs incurred for malaria admissions		Hospital admissions, outpatient visits for malaria
Legesse et al. [60]	LIC	Governmental organization	Child care	Non-RCT	Primary health care units	Integrated community case management (iCCM) program	Region, site, patient	⊠	⊠		⊠		Key indicators of iCCM implementation strength, quality of care, utilization of iCCM services, service			Syndromes treated (malaria, suspected pneumonia, diarrhea, severe acute malnutrition)
Solberg et al. [61]	HIC	Not found	Depression	RCT	Primary care clinics	The IMPACT (Improving Mood: Promoting Access to Collaborative Treatment) model	Site, patient	⊠		⊠	⊠					Depression remission rates, satisfaction with care, work productivity, health status
Sim et al. [62]	MIC	Voluntary/charitable body	HIV	Before-after	Public health facilities	Linked Response (LR) model	Region, site						Coverage			Pregnant women's access to HIV testing and treatment
Munos et al. [63]	LIC	Governmental organization	Child care	Non-RCT	Health districts	iCCM for diarrhea, malaria and pneumonia	Region	⊠	⊠	⊠	⊠					Program targets of mortality and coverage, intensity and quality of program implementation, careseeking.
Singh et al. [64]	MIC	Voluntary/charitable body	Child care	Before-after	Health facilities (health posts, health centers, and hospitals)	Project Fives Alive!	Site	⊠			⊠					Early antenatal care, skilled delivery coverage, underweight infants at child welfare clinics, under-fives

*MIC* middle-income country, *LIC* low-income country, HIC high-income country, *RCT* randomized controlled trial, *CBA* controlled before-and-after, *HIV* human immunodeficiency virus, *DOTS* directly observed treatment, short-course, *YES* youth friendly services, *EMRI* Ethiopian Millennium Rural Initiative, *ART* antiretroviral therapy, *iCCM* integrated community case management, *IMPACT* Improving Mood: Promoting Access to Collaborative Treatment, *LR* linked response

^a^Components of scaling-up strategy: (C_1_) healthcare infrastructure, (C_2_) policy/regulation, (C_3_) financing, (C_4_) human resources, and (C_5_) patient involvement

^b^See Table 2 for more details regarding scaling-up coverage of the targeted units

⊠ means that the scaling-up strategy component was used in the study

The majority of study designs were before-and-after (no control) (*n* = 8) [52–55, 57, 59, 61, 64], followed by six studies with control groups: non-randomized controlled trials (*n* = 4) [51, 56, 60, 63], randomized controlled trials (*n* = 1) [62], and controlled before-and-after (*n* = 1) [58].

Most studies concerned the scaling up of a unique EBP (*n* = 12), while two studies concerned the same EBP (an integrated community case management program) [60, 63].

#### Participants

Targeted units (i.e., intended recipients) were mostly clinical sites (*n* = 11) [52–56, 58–62, 64], followed by patients (*n* = 5) [51, 53, 59, 60, 62], geographical regions (*n* = 4) [51, 60, 61, 63], and health care providers (*n* = 2) [54, 57] (Table 1).

#### Interventions

Components of scaling-up strategies mentioned in the studies were, in order of frequency, those relating to human resources (C_4_) (e.g., policymakers/managers, providers, external medical consultants and community healthcare workers) (*n* = 12) [51, 53–60, 62–64], components relating to healthcare infrastructure (C_1_) (e.g., new buildings, linkages between different clinical sites) (*n* = 6) [55, 58, 60, 62–64], components related to changes in policy/regulation (C_2_) (*n* = 5) [52, 56, 59, 60, 63], and components related to financing (C_3_) (e.g., paying bonuses to healthcare workers) (*n* = 2) [62, 63]. As reported in Table 1, eight studies mentioned several components of a multifaceted scaling-up strategy [55, 56, 58–60, 62–64] (ranging from two to four components), while five studies [51–54, 57] only mentioned one component. Six studies [55, 58, 60–62, 64] reported on scaling up an EBP across different settings in a phased manner (i.e., horizontal scaling-up strategy). Eight studies did not mention whether the scaling up was horizontal (phased) or vertical (simultaneously to a whole system).

#### Outcomes

Only five studies [51, 55, 57, 60, 61] reported scaling-up process outcomes: three studies [51, 57, 61] reported coverage of the targeted units, one study [55] reported on costs, and two studies [57, 60] reported on other process measures (Table 1).

Three studies [51, 57, 61] reported coverage comprehensively, i.e., number of targeted units that received the EBP over all those targeted. Respectively, these scaling-up coverages were achieved after 30, 22, and 57 months of the scaling-up process (Table 2). As detailed in Table 2, information on coverage was unavailable or incomplete in 11 studies. Seven studies reported only the number of units targeted (denominator) but not the number covered (numerator) [52, 55, 56, 58, 59, 63, 64]; three reported the number of patients/providers covered and the number of *sites* targeted [53, 54, 62]; one reported the number of sites covered but the number of *patients* targeted [60].

**Table 2:** Reported coverage measures (*n* = 14)

Reference in chronological order	Numerator (*n*)				Denominator (*d*)				Coverage (*n*/*d*) (%)				Timeframe of the scaling-up process	Reported successful coverage			Reported impact on main health outcomes			Used framework	Used framework
	Regions	Sites	Providers	Patients	Regions	Sites	Providers	Patients	Regions	Sites	Providers	Patients	Months	+	–	Unclear	+	–	Unclear	Name	No
Frieden et al. [51]								10,000,000				74	30	⊠			⊠^g^				⊠
Price et al. [52]						30							6			⊠	⊠^g^				⊠
Mutevedzi et al. [53]				5719		16							48	⊠				⊠^g^			⊠
Renju et al. [54]			429		4	177							39	⊠			⊠^g^				⊠
Curry et al. [55]						30				^e^			18	⊠			⊠				⊠
Goetz et al. [56]						15^c^							12	⊠			⊠^g^				⊠
Li et al. [57]			157			63	323				49		22	⊠			⊠			RE-AIM	
Miyano et al. [58]						8							≤ 36	⊠			⊠^g^				⊠
Comfort et al. [59]						49^d^		400,335^d^					≤ 72	⊠			⊠				⊠
Legesse et al. [60]		13,500		^a^	4			70,000,000					31	⊠					⊠		⊠
Solberg et al. [61]				2348		75							63		⊠			⊠^g^			⊠
Sim et al. [62]	74	956			77	1004			96	95			57	⊠^g^			⊠				⊠
Munos et al. [63]					9							^f^	35		⊠			⊠^g^			⊠
Singh et al. [64]				^b^		744							43			⊠	⊠^g^				⊠

*RE-AIM* Reach, Effectiveness, Adoption, Implementation, and Maintenance

^a^Authors reported that the EBP reached an estimated 10,230,450 under-5s

^b^Authors provided coverage information for some indicators (*d* = 744 sites): early antenatal care (*n* = 11,671 patients), skilled delivery (*n* = 9573 patients), and underweight in infants (*n* = 7685 patients)

^c^Sum of seven and eight facilities of two arms of this trial

^d^Sum of 13 rural health centers (covering 160,000 persons), 19 primary health centers and 17 rural health centers (covering 240,335 persons)

^e^Authors provided information on achieved coverage for some indicators (*d* = 30 sites): access to water (from 27 to 100%), access to electricity (from 73 to 97%), and health center staffing (from 75 to 90%)

^f^Authors provided information on program targets, baseline, and achieved levels of mortality and coverage: under-five mortality rate (target, 82.5 deaths per 1000; baseline, 110 deaths per 1000; achieved, 103 deaths per 1000), ≥ 4 antenatal care visits (targeted, 80%; baseline, 45%; achieved, 44%), intermittent preventive treatment of malaria in pregnancy (targeted, 70%; baseline, 44%; achieved, 39%), skilled birth attendance (targeted, 60%; baseline, 80%; achieved, 73%), cesarian section (targeted, 2%; baseline, 3%; achieved, 2%), early initiation of breast-feeding (targeted, 40%; baseline, 25%; achieved, 26%), postpartum vitamin A (targeted, 60%; baseline, 50%; achieved, 57%), artemisinin combination therapy for fever (targeted, 70%; baseline, 27%; achieved, 23%), antibiotics for pneumonia (targeted, 60%; baseline, 30%; achieved, 16%), oral rehydration therapy + continued feeding (targeted, 60%; baseline, 65%; achieved, 64%), insecticide-treated bednets (targeted, 70%; baseline, 51%; achieved, 92%), exclusive breast-feeding (targeted, 20%; baseline, 35%; achieved, 42%), vitamin A supplementation (targeted, 90%; baseline, 89%; achieved, 93%)

^g^Quantitatively reported successful coverage or impact of scaling-up strategy

⊠ is a checkmark for this item

Beyond scaling-up process measures, patient outcomes were the most reported outcomes (*n* = 13) [51–53, 55–64], followed by health system outcomes (*n* = 3) [55, 56, 60] and provider outcomes (*n* = 2) [54, 56] (Table 1).

Only one included study [57] used a model to assess the impact of the scaling-up strategy, namely the “Reach, Effectiveness, Adoption, Implementation and Maintenance” (RE-AIM) model.

### Success of the scaling-up strategies

One study [61] quantitatively reported successful coverage, i.e., coverage of 80% was achieved (Table 2). Of the 13 remaining studies, nine qualitatively reported successful coverage [51, 53–60] (e.g., “Ethiopia’s iCCM strategy has accomplished much and contributed to national and global learning” [60]), while two reported that scaling up did not succeed [62, 63] (e.g., “The ‘Rapid Scale-Up’ did not result in coverage increases (of intervention) or mortality reductions in Burkina Faso” [63]) and two were unclear [52, 64].

In terms of the main outcomes of studies (provider/patient outcomes), six [51, 52, 54, 56, 58, 64] quantitatively reported a favorable impact (i.e., using statistical methods), while three studies [53, 62, 63] quantitatively reported no impact. Of the five remaining studies, four [55, 57, 59, 61] qualitatively reported a favorable impact (e.g., “The number of pregnant women tested for HIV increased from 41,800 of 67,924 (61.5%) in 2009 to 269,935 of 361,655 (70.7%) in 2012” [61]). In one study [60], information about impact was unclear.

No study quantitatively reported achieving coverage of 80% in combination with a favorable impact on its main outcomes.

### Quality assessment of studies

Our assessment of the 14 included studies using the Quality Assessment Tool for Quantitative Studies resulted in a strong rating for six studies [52, 53, 58, 59, 61, 63], a moderate rating for two studies [56, 62], and a weak rating for six studies [51, 54, 55, 57, 60, 64] (Additional file 5).

### Data validation with authors of studies

We contacted authors of included studies to validate our data extraction. Overall, we received eight responses (57.1%) from corresponding authors.

## Discussion

To the best of our knowledge, our review is the first to explore elements of strategies for scaling up EBPs in primary care settings. We found few studies assessing the impact of scaling-up strategies of EBPs in primary care. Most were conducted in low- and middle-income countries. Most were funded by international and national governmental organizations. The most represented clinical area was infectious diseases followed by newborn/child care, depression, and preventing seniors’ falls. Study designs were mostly before-and-after studies, without control. Clinical sites (e.g., hospitals, health posts, community health centers) were the most frequently targeted units. The component of the scaling-up strategy most mentioned was human resource-related. Very few studies provided information on the scaling-up metrics, i.e., coverage of the units they targeted. While several studies reported on the success of the scaled-up EBP in terms of patient/provider outcomes, no studies quantitatively reported on the success of the scaling-up strategy itself. These results lead us to make the following observations.

First, only a small number of studies were identified. Although this may not be surprising giving the emerging nature of the field and its dispersal across many disciplines, it is possible that the search strategy did not identify all relevant studies. Despite the inclusion of a broad range of databases, scale up and spread are ill-defined and under-theorized concepts [14], and there is a lack of consensus within the field regarding terminology. The development of a validated search filter for “scale up” of EBPs would be of particular value for future reviews in the field to balance the sensitivity and specificity of search strategies and reduce the likelihood of omitting pertinent research.

Second, the majority of studies were undertaken in low- and middle-income countries and focused on EBPs tackling infectious diseases. The overrepresentation of low- and middle-income countries in our review is consistent with previous work [15]. This could be explained by the burden of care that these countries were facing at the time which, due to rapid spread of infectious diseases, was quickly increasing and urgently required the scaling up of specific EBPs to address these threats to their population [65–68]. In addition, given the financial constraints faced by low- and middle-income countries, it would appear reasonable to scale up existing EBPs rather than spend more resources in developing new EBPs. In addition, low- and middle-income countries often incentivise health systems research that promotes global health equity [69]. Moreover, many primary care EBPs in low- and middle-income countries originate at very local levels, often supported by non-governmental organizations and external funding. Many countries attempt to eliminate their infectious disease burden by scaling up these small-scale EBPs to achieve broad impact at the national level [67]. The scaling up, too, therefore often involves foreign nationals and world leaders. For example, through the Millennium Development Goals [70], the World Health Organization is working with world leaders on scaling up initiatives to reduce child mortality, improve maternal health, and combat HIV/AIDS, malaria, and tuberculosis, clinical areas identified in almost all our included studies.

Third, we found poor representation of high-income countries in studies assessing the impact of strategies to scale up EBPs in primary care settings. In high-income economies, much funding of health research has focused on development and testing of original and innovative interventions, and less on the scaling up of these interventions [66, 71]. High-income countries also face a very different burden of care, mostly non-communicable chronic diseases. These diseases and a growing proportion of elderly citizens increasingly drive the demand for healthcare, and there is a new urgency to scale up effective EBPs that address these concerns in the hope of saving costs [65, 68, 72–74]. High-income countries are therefore taking a new interest in scaling-up research [37, 71, 74–76]. Based on the results of this review, scaling-up strategies developed in low- and middle-income countries may now be in a position to inform the scaling up of EBPs in high-income countries [66, 77]. This is a reversal long needed to increase the capacity of low- and middle-income countries to play a major role in the field of implementation science [78], although transferability of strategies to different settings will continue to be a challenge [25, 79]. This is an area that needs further investigation [79].

Fourth, we noted vast inconsistencies in the reporting of scaling up and its critical components. Although some studies were rated high quality (regarding what was done in terms of selection, design, and data collection, for example), relevant information on the scaling-up process they used was unavailable. For example, scaling-up strategies were poorly defined, and most studies focused more on the EBP itself. As the science of scaling up is a relatively new field, there is little guidance on how to assess or report on scaling-up strategies. Overall, studies did not attempt to provide the information needed to foster the use or replication of their scaling-up strategies [80]. It has been found that overall, more than half of clinical treatments are not beneficial [81], and so there is clearly no point scaling up the majority of clinical practices. If few clinical practices have beneficial effects under controlled delivery conditions, even fewer are effective under real-world conditions [9, 27, 81]. Problems and difficulties with scaling up even proven clinical practices suggest that it might be beneficial to identify phases as well as components of scaling up. The current literature [16, 27] highlights four phases that could be documented on a more systematic basis: (1) assessment of scalability; (2) development of the scaling plan; (3) preparation of material, financial, and human resources; and (4) scaling up of the EBP. While the “Standards for Reporting Implementation Studies” (StaRI) could be a starting point, they do not cover core components of scaling-up strategies [80].

Fifth, we found very little measurable evidence regarding the success of the scaling-up strategies in the studies we reviewed. This could be explained by the lack of consensus on scaling-up outcomes. It could also be because of the heterogeneity of EBPs, leading to an assumption among researchers that each EBP must be scaled up in a different way, and there can be no single set of procedures or measures. We suggest that proper reporting on scaling up would require both a denominator (number of targeted units) and a numerator (number of units covered by the EBP) [33], in combination with impact measurements. Central to evaluation of the success of scale-up initiatives is the extent to which an EBP achieved its intended benefit on the targeted populations [31, 43, 82, 83]. Coverage of the targeted population is therefore a key indicator for measuring this success [33, 43, 83]. For EBPs to have a substantial impact, it is also necessary to have a large enough population coverage over a sustained period [83]. While there is no consensus yet on a threshold of coverage that would indicate success, several international scaling-up initiatives and a large implementation review have identified 80% as a reasonable target coverage [43–50]. The power of scaling up is in its ability to maximize the benefits of EBPs, i.e., produce a numerator substantial enough [33, 83].

Our study has limitations. First, we cannot assume that we found all potentially eligible studies. However, we consulted many literature sources and implementation experts in order to substantially reduce this limitation. Second, little clear information on the scaling-up process itself was reported in the published materials, and it was difficult to clearly draw a distinction between the components of the EBP and the components of the scaling-up strategy. However, we invited all corresponding authors of the included studies to validate the extracted data and provide any missing information. Although we did not receive a perfect response rate, more than half of them responded. Finally, considering the low number of studies included, this review is limited in its capacity to provide clear guidance on scaling-up strategies. The review was triggered by the needs of Canadian policymakers who are proceeding with scaling up effective healthcare practices, but find there is little guidance on strategy, methods, and evaluation measures. In spite of the few studies in our review, we believe it was urgent to “get the ball rolling” by bringing together scaling-up studies in primary care from among the many disciplines in which they can be found; show their vast inconsistency in reporting on scaling-up strategies, methods, and measures; and lay the groundwork for future studies.

## Conclusions

We found few studies assessing the impact of scaling-up strategies of EBPs in primary care in terms of coverage of the targeted units. Most were conducted in low- and middle-income countries. The most represented clinical area was infectious diseases followed by newborn/child care, depression, and prevention of seniors’ falls. The component of a scaling up strategy most frequently reported was related to human resources. As very few studies provided a measure of the coverage of the scaled-up intervention, it is uncertain whether their scaling up strategies were effective. The science of scaling up EBPs in primary care is young and future initiatives should include the development of specific reporting guidelines and minimal consensus on the metrics of scaling-up studies.

## Additional files

Additional file 1: Review inclusion and exclusion criteria. (DOCX 44 kb)

Additional file 2: Search strategy in MEDLINE (Ovid), August 30, 2016. (DOCX 44 kb)

Additional file 3: Gray literature search. (DOCX 47 kb)

Additional file 4: Email sent regarding validation of data extraction. (DOCX 43 kb)

Additional file 5: Quality assessment of 14 included studies using the Effective Public Health Practice Project tool. (DOCX 14 kb)

## Abbreviations

EBP: Evidence-based practice
EPOC: Effective Practice and Organisation of Care
iCCM: Integrated community case management
PICO: Participant, intervention, comparator, outcome
PRISMA: Preferred Reporting Items for Systematic Reviews and Meta-Analyses
RE-AIM: Reach, Effectiveness, Adoption, Implementation and Maintenance
StaRI: Standards for Reporting Implementation Studies.
