# Supplementary material for: Effective strategies for scaling up evidence-based practices in primary care: a systematic review
Source: Implement Sci. 2017 Nov 22;12:139. doi: 10.1186/s13012-017-0672-y (PMC5700621; doi:10.1186/s13012-017-0672-y)
Supplement: Supplementary file 1 — Review inclusion and exclusion criteria. (DOCX 44 kb) [file 13012_2017_672_MOESM1_ESM.docx]

**Additional file 1:** Review inclusion and exclusion criteria

| **Criteria** | **Inclusion** | **Exclusion** |
| --- | --- | --- |
| Type of document | - Original articles - Evaluation and research report - Government documents | - Editorial - Comment and opinion letter - Abstract of conference - Protocol |
| Study design | - Randomized controlled trial - Non-randomized controlled trial - Controlled before-and-after - Before-and-after (i.e., without control group) - Interrupted time series | - Literature review - Meta-analysis |
| Setting | - Primary care | - Public health |
| Intervention | - Scaling up of an evidence-based practice - Spread of an evidence-based practice - Evaluation of scaling up or spread | - Not evidence-based practice |
