# Supplementary material for: Effective strategies for scaling up evidence-based practices in primary care: a systematic review
Source: Implement Sci. 2017 Nov 22;12:139. doi: 10.1186/s13012-017-0672-y (PMC5700621; doi:10.1186/s13012-017-0672-y)
Supplement: Supplementary file 2 — Search strategy in MEDLINE (Ovid), August 30, 2016. (DOCX 44 kb) [file 13012_2017_672_MOESM2_ESM.docx]

**Additional file 2**: Search strategy in Medline (Ovid), August 30th 2016

| **#** | **Searches** | **Findings** |
| --- | --- | --- |
| 1 | ("scaling up" or "scaled up" or "scale up" or "up-scaling" or "upscaling").ti,ab. |  |
| 2 | (scalability or scalable or "at scale").ti,ab. |  |
| 3 | (spread adj5 (innovation* OR intervention* OR technolog* OR practice OR care)).ti,ab. |  |
| 4 | ((bring* or brought or taking or take* or increas* or going or implement*) adj5 scale)).ti,ab. |  |
| 5 | or/1-4 | 33 421 |
| 6 | (primary adj3 care).ti,ab. |  |
| 7 | ("primary healthcare" or "primary health" or "first line").ti,ab. |  |
| 8 | ((family or general or group) adj2 (doctor or doctors or physician* or practice* or practitioner* or medicine)).ti,ab. |  |
| 9 | (rural adj3 (physician* or practice or practitioner*)).ti,ab. |  |
| 10 | generalist*.ti,ab. |  |
| 11 | (ambulatory adj2 (care or clinic)).ti,ab. |  |
| 12 | (health adj3 (center* or centre*)).ti,ab. |  |
| 13 | consult*.ti,ab. |  |
| 14 | (visit* adj3 (clinic* or care or outpatient)).ti,ab. |  |
| 15 | (community adj3 (care or worker* or service*)).ti,ab. |  |
| 16 | Primary Health Care/ |  |
| 17 | General Practice/ or Family Practice/ or Physicians, Family/ or General Practitioners/ or Physicians, Primary Care/ or Group practice/ |  |
| 18 | Ambulatory Care/ |  |
| 19 | Community Health Services/ OR Community Health Centers/ or Community Mental Health Services/ or Community Mental Health Centers/ |  |
| 20 | or/6-19 | 545 540 |
| 21 | (random* or nonrandom*).ti,ab. |  |
| 22 | ((single or double or triple) adj3 (blind$3 or mask$3)).ti,ab. |  |
| 23 | (control* adj3 trial* ).ti,ab. |  |
| 24 | ((comparison or control) adj3 group).ti,ab. |  |
| 25 | quasiexperimental.ti,ab. |  |
| 26 | ((quasi or pseudo) adj3 experimental).ti,ab. |  |
| 27 | (before adj5 after).ti,ab. |  |
| 28 | (pre adj5 post).ti,ab. |  |
| 29 | (pretest adj5 posttest).ti,ab. |  |
| 30 | ("time series" adj5 interrupted).ti,ab. |  |
| 31 | ("repeated measure" OR "repeated measures").ti,ab. |  |
| 32 | longitudinal.ti,ab. |  |
| 33 | ((evaluat* or compar*) adj3 study).ti,ab. |  |
| 34 | Randomized Controlled Trial/ or Randomized Controlled Trials as Topic/ |  |
| 35 | Random Allocation/ or Double-Blind Method/ or Single-Blind Method/ or Control groups/ |  |
| 36 | Comparative Study/ |  |
| 37 | Clinical Trial as Topic/ or Controlled Clinical Trial as Topic/ |  |
| 38 | Controlled Before-After Studies/ |  |
| 39 | Longitudinal Studies/ |  |
| 40 | Interrupted Time Series Analysis/ |  |
| 41 | Evaluation Studies/ or Evaluation Studies as Topic/ or Validation Studies/ |  |
| 42 | ("Randomized Controlled Trial" or "Clinical Trial" or "Controlled Clinical Trial" or "Comparative Study" or "Evaluation Studies").pt. |  |
| 43 | or/21-42 | 4 176 202 |
| **44** | **5 and 20 and 43** | **662** |
