# Supplementary material for: Effective strategies for scaling up evidence-based practices in primary care: a systematic review
Source: Implement Sci. 2017 Nov 22;12:139. doi: 10.1186/s13012-017-0672-y (PMC5700621; doi:10.1186/s13012-017-0672-y)
Supplement: Supplementary file 3 — Gray literature search. (DOCX 47 kb) [file 13012_2017_672_MOESM3_ESM.docx]

**Additional file 3:** Grey literature search

| Website searched | Search date | Keywords |
| --- | --- | --- |
| Google | 20-06-2016 | scaling up (primary care OR first line) |
|  | 06-07-2016 | ("mise à l'échelle" OR "accroissement d'échelle") ("soins primaires" OR "première ligne") filetype:pdf |
|  | 02-08-2016 | "passage à l'échelle" ("soins primaires" OR "première ligne") |
| Google Scholar | 27-06-2016 | scaling up (primary care OR first line) |
|  | 06-07-2016 | ("mise à l'échelle" OR "accroissement d'échelle") ("soins primaires" OR "première ligne") |
|  | 02-08-2016 | "passage à l'échelle" ("soins primaires" OR "première ligne") |
| [Mednar](http://mednar.com/mednar/desktop/en/green/search.html) | 27-06-2016 | "scaling up" ("primary care" OR "first line") |
| [NHS Evidence](https://www.evidence.nhs.uk/) | 27-06-2016 | "scaling up" ("primary care" OR "first line") |
| [Grey Literature Report](http://www.greylit.org/) | 27-06-2016 | "scaling up" |
| [Open Grey](http://www.opengrey.eu/) | 27-06-2016 | "scaling up" |
| [EuroScan](https://www.euroscan.org/) | 27-06-2016 | "scaling up" |
| [Banque de données en santé publique](http://www.bdsp.ehesp.fr/) | 01-08-2016 | "mise à l’échelle"  "accroissement d’échelle"  "passage à l’échelle" |
| [Érudit](https://www.erudit.org/en/) | 01-08-2016 | All fields (except full text) : "mise à l'échelle" OR "passage à l'échelle" OR "accroissement d'échelle" |
| [Réseau Santécom](http://catalogue.santecom.qc.ca/) | 27-06-2016 | "mise à l'échelle"  "passage à l’échelle"  "accroissement d’échelle" |
| [Institut National d'Excellence en Santé et Services Sociaux](https://www.inesss.qc.ca/publications/publications.html) | 27-06-2016 | échelle |
| [Canadian Evaluation Society](http://evaluationcanada.ca/) | 10-08-2016 | scale |
| [Canadian Agency for Drugs and Technologies in Health](https://www.cadth.ca/reports?keywords=&result_type%5b0%5d=report&sort=field_date%3Avalue-desc&amount_per_page=10&email=&page=1) | 27-06-2016 | "scaling up"  "mise à l'échelle"  "passage à l’échelle"  "accroissement d’échelle" |
| [Health Canada](http://www.hc-sc.gc.ca/) | 27-06-2016 | "scaling up"  "mise à l'échelle"  "passage à l’échelle"  "accroissement d’échelle" |
| [International Network of Agencies for Health Technology Assessment](http://www.inahta.org/) | 27-06-2016 | Scaling up |
| [World Health Organization](http://search.who.int/search?q=&ie=utf8&site=who&client=_en_r&proxystylesheet=_en_r&output=xml_no_dtd&oe=utf8&getfields=doctype) | 06-07 2016 | "scaling up" ("primary care" OR "first line")  Filter : PDF |
| [National Institute for Health and Care Excellence](https://www.nice.org.uk/) | 06-07 2016 | Scaling up |
| [Joanna Briggs Institute](http://joannabriggs.org/) | 27-06-2016 | Scaling up |
| [CIHR Funding decisions database](http://webapps.cihr-irsc.gc.ca/cfdd/db_search?p_language=E) | 18-08-16 | Scaling up  Scale up  Scaled up  At scale  To scale  Passage à l’échelle |
| [NIH Reporter](https://projectreporter.nih.gov/reporter.cfm) | 05-12-2016 | "scaling up" search in Projects, Limit Project search to Project Title (Fiscal year all)  "scale up"  "scaled up"  "going to scale"  "up scaling" |
| ProQuest dissertations and theses | 01-08-2016 | all("scaling up" OR "scale up" OR "scaled up" OR "up scaling" OR "going to scale") AND all(("primary care" OR "first line" OR "frontline")) |
| [Theses Canada](http://amicus.collectionscanada.gc.ca/thesescanada-bin/Main/AdvSearch?coll=18&l=0&v=1) | 01-08-2016 | all fields : "primary care" or "first line" and "scaling up"  all fields : "primary care" or "first line" and "scale up"  all fields : "primary care" or "first line" and "scaled up"  "soins primaires" or "première ligne" and "mise à l’échelle"  "soins primaires" or "première ligne" and "accroissement d’échelle"  "passage à l’échelle" |
| [DART-Europe](http://www.dart-europe.eu/basic-search.php) | 01-08-2016 | "scaling up" |
| [Clinicaltrials.gov](https://clinicaltrials.gov/) | 01-08-2016 | "scaling up" |
| [EU Clinical Trials Register](https://www.clinicaltrialsregister.eu/ctr-search/search) | 01-08-2016 | "scaling up"  "scale up"  "scaled up"  "going to scale"  "up scaling" |
| [Australian New Zealand Clinical Trials Registry](file:///C:\Users\beal3025\AppData\Local\Microsoft\Windows\Temporary%20Internet%20Files\Content.Outlook\Q460ZQG6\ANZCTR) | 01-08-2016 | "scaling up" |
| [International Clinical Trials Registry Platform](http://apps.who.int/trialsearch/AdvSearch.aspx) | 10-08-2016 | "scaling up" in Title |
| [UMIN Clinical Trials Registry](http://www.umin.ac.jp/ctr/) | 02-08-2016 | "scaling up"  "scale up"  "scaled up"  "going to scale"  "up scaling" |
