# Supplementary material for: Effective strategies for scaling up evidence-based practices in primary care: a systematic review
Source: Implement Sci. 2017 Nov 22;12:139. doi: 10.1186/s13012-017-0672-y (PMC5700621; doi:10.1186/s13012-017-0672-y)
Supplement: Supplementary file 5 — Quality assessment of 14 included studies using the Effective Public Health Practice Project tool. (DOCX 14 kb) [file 13012_2017_672_MOESM5_ESM.docx]

**Additional file 5**: Quality assessment of 14 included studies using the Effective Public Health Practice Project tool

| **Reference in chronological order** | **Selection bias** | **Study design** | **Confounders** | **Blinding** | **Data collection method** | **Withdrawals and dropouts** | **Global rating** |
| --- | --- | --- | --- | --- | --- | --- | --- |
| Frieden et al. 2003 | Moderate | Strong | Weak | Strong | Weak | Weak | Weak |
| Price et al. 2009 | Moderate | Moderate | Strong | Strong | Strong | Moderate | Strong |
| Mutevedzi et al. 2010 | Moderate | Moderate | Strong | Strong | Strong | Moderate | Strong |
| Renju et al. 2010 | Moderate | Moderate | Weak | Moderate | Weak | Moderate | Weak |
| Curry et al. 2013 | Moderate | Moderate | Strong | Moderate | Weak | Weak | Weak |
| Goetz et al. 2013 | Moderate | Strong | Weak | Strong | Strong | Strong | Moderate |
| Li et al. 2013 | Moderate | Moderate | Strong | Weak | Weak | Moderate | Weak |
| Miyano et al. 2013 | Moderate | Strong | Strong | Moderate | Strong | Moderate | Strong |
| Comfort et al. 2014 | Moderate | Moderate | Strong | Moderate | Moderate | Moderate | Strong |
| Legesse et al. 2014 | Weak | Strong | Weak | Moderate | Weak | Weak | Weak |
| Solberg et al. 2015 | Weak | Strong | Strong | Moderate | Strong | Moderate | Moderate |
| Sim et al. 2015 | Strong | Moderate | Strong | Moderate | Strong | Moderate | Strong |
| Munos et al. 2016 | Moderate | Strong | Strong | Strong | Moderate | Moderate | Strong |
| Singh et al. 2016 | Weak | Moderate | Strong | Strong | Strong | Weak | Weak |
