# Supplementary material for: Effective strategies for scaling up evidence-based practices in primary care: a systematic review
Source: Implement Sci. 2017 Nov 22;12:139. doi: 10.1186/s13012-017-0672-y (PMC5700621; doi:10.1186/s13012-017-0672-y)
Supplement: Supplementary file 4 — Email sent regarding validation of data extraction. (DOCX 43 kb) [file 13012_2017_672_MOESM4_ESM.docx]

**Additional file 4:** Sent email regarding validation of data extraction

Dear Dr. “name of corresponding and/or first author”,

Hoping this email finds you well.

I am a postdoctoral researcher in Knowledge Translation under the supervision of Drs France Légaré and Annie LeBlanc. We are currently conducting a systematic review entitled "Effective interventions for scaling up evidence-based practices in primary care: a systematic review".

Your article titled “Title of publication” has been identified for inclusion in our review. We would be most grateful if you could validate the extraction we have performed of your study, correcting any inaccuracies and providing any missing information (see attached document). This should not take you more than a few minutes of your time and will ensure appropriate representation of your work.

We would appreciate if you could let us know by **February 15^th^ 2017**. Please don’t hesitate to contact us for further details.

Thank you very much for your help.
